# Interrelationships between Refined Carbohydrates, Periodontal Diseases, and Cognitive Decline: A Narrative Review

**DOI:** 10.1016/j.advnut.2026.100657

**Published:** 2026-05-14

**Authors:** Daisy Recchia, Michel Raymond, Claire Berticat, Sylvaine Artero

**Affiliations:** 1 ISEM (Institut des Sciences de l'Évolution de Montpellier), CNRS (Centre National de la Recherche Scientifique), IRD (Institut de Recherche pour le Développement), University of Montpellier, Montpellier, France; 2 IGF (Institut de Génomique Fonctionnelle), CNRS (Centre National de la Recherche Scientifique), INSERM (Institut National de la Santé et de la Recherche Médicale), University of Montpellier, Montpellier, France

**Keywords:** sugars, glycemic carbohydrates, glycaemic carbohydrates, periodontitis, dementia, carbohydrate quality, gingivitis, cognition, Alzheimer’s disease, public health

## Abstract

Accumulating studies suggest that carbohydrate quality influences both oral and cognitive health. Diets rich in refined carbohydrates promote insulin resistance, oxidative stress, inflammation, and disrupt oral microbiota. These changes contribute to periodontal disease and neurodegeneration. Periodontitis is associated with cognitive decline via inflammation, bacterial translocation, and blood–brain barrier disruption. Although less extensively studied, reverse pathways may also operate. Cognitive decline may alter dietary behavior through neurohormonal changes, increasing preference for sweeter foods, and promoting periodontal disease by impairing motor functions essential for oral hygiene. Periodontitis may further shift dietary choices toward softer, more processed foods through tooth loss, oral pain, and chewing difficulties. These interactions may reinforce a cycle linking diet, oral health, and cognition.

To date, no study has comprehensively examined the interrelationships between carbohydrate quality, periodontal disease, and cognitive decline within a unified framework. This narrative review aimed to address this gap by synthesizing findings on each bidirectional dyadic association, examining terminology and methodological approaches, and discussing shared mechanisms and biological pathways. Through this work, insulin resistance, chronic inflammation, oxidative stress, and microbiota dysbiosis were identified as shared integrative pathways, and key research gaps were highlighted to guide future research and inform prevention strategies for aging populations.

**Statements of Significance**

This narrative review offers a novel perspective by integrating 3 domains never before examined together: refined carbohydrate intake, periodontal health, and cognitive decline. By outlining the biological and behavioral pathways that link excessive refined carbohydrate consumption to both oral and cognitive health, and by identifying key research gaps, our review provides a strong foundation for developing more integrated approaches to dementia risk assessment and prevention.

## Introduction

The global burden of cognitive decline and dementia is rising rapidly, with projections estimating over 150 million cases worldwide by 2050 [1]. Although aging remains the primary risk factor, modifiable lifestyle factors, such as diet, are increasingly recognized for their potential role in prevention [2]. Emerging evidence also highlights the importance of oral health, which is closely linked to both dietary habits [3] and cognitive health [4]. Oral diseases affect ∼3.5 billion people worldwide, with severe periodontal disease impacting ∼10% of the global population [5]. Among dietary components, carbohydrate quality, specifically lower intake of refined carbohydrates and higher intake of whole grains (i.e., nonrefined carbohydrates) and dietary fibers, has emerged as a key contributor to systemic and brain health [[6], [7], [8]].

Refined carbohydrates, encompassing refined sugars (i.e., free sugars) and refined grains, are rapidly digested and result in pronounced postprandial glycemic and insulinemic responses [[9], [10], [11], [12]]. Refined carbohydrates include fructose-rich products, such as high-fructose corn syrup, which, despite having a lower glycemic index, may raise dementia risk by promoting insulin resistance [13]. Consumption of refined carbohydrates, such as white bread, sugary beverages, and processed snacks, has increased globally over the past decades, especially in industrialized and transitioning countries [14]. This dietary trend has been linked to a rise in metabolic conditions such as obesity, insulin resistance, and type 2 diabetes, which are also risk factors for cognitive impairment [15].

High intake of refined carbohydrates may contribute to cognitive decline through mechanisms involving oxidative stress, insulin resistance, and systemic inflammation [11]. Additionally, such diets may alter the gut–oral–brain axis by promoting dysbiosis and inflammatory responses, contributing to the onset and progression of periodontal diseases [16]. These inflammatory oral conditions, primarily gingivitis and periodontitis, are increasingly viewed not only as local disorders but also as systemic inflammatory diseases with potential neurological consequences [17]. The number of studies published on the associations between periodontitis and cognitive decline is growing considerably [[17], [18], [19], [20]], suggesting several potential pathways: translocation of oral pathogens [e.g., *Porphyromonas gingivalis (P. gingivalis*)] [18], neuroinflammatory signaling [21], potential involvement of microRNAs [22,23], and shared vascular [24,25] or metabolic risk factors, such as type 2 diabetes [26].

Despite increasing findings supporting pairwise associations among refined carbohydrate intake, periodontal disease, and cognitive decline, these relationships have not yet been examined within an integrated conceptual framework. This fragmented approach limits a comprehensive understanding of shared biological mechanisms and potential mediating pathways. The present narrative review, therefore, aimed to critically synthesize findings from epidemiological and review studies addressing each dyadic association, with particular attention to bidirectional relationships. It further aimed to evaluate current terminology and methodological approaches, discuss proposed mechanisms and shared biological pathways, and identify key knowledge gaps to inform future research and prevention strategies targeting aging populations.

### Terminology and assessment methods

Clarifying how refined carbohydrates, periodontal diseases, and cognitive decline are defined and measured is essential to understanding the complex relationships between these 3 factors. Inconsistent terminology and methodological approaches likely contribute to the heterogeneity observed in the literature. This section presents key definitions and measurements for each of the 3 factors examined in this review.

#### Refined carbohydrates

Carbohydrate terminology used in nutrition research is highly inconsistent [[27], [28], [29]]. Commonly used terms, such *as total sugars, free sugars,* and *added sugars,* capture only mono- and disaccharides, excluding starches, even though evidence suggests that all digestible carbohydrates, including starches, can influence glycemic response, inflammation, and microbiota composition [11]. The term *glycemic carbohydrates* referred to digestible sugars and starches that raise postprandial blood glucose concentrations, typically measured using the glycemic index or glycemic load [30], with some foods being high glycemic (rapidly raising glucose) and others low glycemic (causing slower, smaller increases). However, this metric has limitations, as some fructose-rich foods (e.g., high-fructose corn syrup, fruit juice, agave, and maple syrup) produce low-glycemic responses yet still contribute to hepatic insulin resistance and metabolic dysfunction [31].

To capture both refined sugars and refined grains, we chose the term refined carbohydrates. However, to our knowledge, there is no universally accepted definition for refined carbohydrates in the current literature. Therefore, we defined *refined carbohydrates* as foods containing carbohydrates processed to remove intrinsic fiber, micronutrients, and/or structural components of the original food matrix, resulting in rapid digestibility and reduced nutrient density for this review. This includes *refined grains*, which are grains processed to remove bran, germ, fiber, and micronutrients [32], and *refined sugars*, namely foods rich in free sugars which is defined by the WHO as all mono- and disaccharides (e.g., glucose, sucrose) added to foods and beverages by the manufacturer, cook, or consumer, as well as sugars naturally present in honey, syrups, fruit juices, and fruit juice concentrates [33].

*Refined carbohydrates* align with the emerging concept of *low carbohydrate quality*, characterized by low fiber and whole-grain intake, high-glycemic index, and high free sugar content [[34], [35], [36]]. Refined carbohydrates were chosen in this review for their specificity in capturing metabolic risks relevant to both periodontal and cognitive outcomes.

##### Measurement approaches used in literature

Links between carbohydrate quality and periodontal or cognitive outcomes are typically explored using self-reported dietary assessment tools such as food frequency questionnaires, 24-h recalls, or dietary records to assess carbohydrate intake. These methods estimate exposures such as total, free [37], or added sugar intake (e.g., percentage of calories) [38,39], sugary food intake (e.g., chocolates, cakes, and sticky sweets) [40], sugar-sweetened beverage (SSB) intake [41,42], or, more specifically, glycemic load at the level of individual meals [43,44]. Other studies estimate starch intake (e.g., grams per day, percentage of calories, or starch type) [45], nonstarch polysaccharides intake [46], or carbohydrate-to-fiber ratios [47]. These measures vary in precision and remain proxies for the true metabolic effects of carbohydrate quality or refined carbohydrate intake.

Although less common, biomarkers such as postprandial and fasting glucose or insulin concentrations, glycated hemoglobin (HbA1c, reflecting mean glycemia over 8‒12 wk), and insulin resistance estimates (e.g., HOMA-IR and triglyceride-glucose index) have also been used to approximate glycemic exposure in relation to periodontal health [48,49] and cognitive decline [50]. Although these measures offer objective assessment, they remain limited by their temporal specificity, inter-individual variability, and potential confounding factors such as physical activity, smoking, and sleep quality, which independently influence these biomarkers [51,52].

Overall, the lack of uniform definitions and consistent measurement approaches for refined carbohydrates remains a major challenge, underscoring the need for harmonization across epidemiologic studies.

#### Periodontal diseases

In periodontal research, gingivitis and periodontitis are the 2 most commonly distinguished conditions. Gingivitis is a reversible inflammation of the gingiva associated with bacterial plaque, whereas periodontitis involves chronic inflammation, attachment loss, and pocket formation, and eventually may lead to alveolar bone loss and tooth loss [53].

Assessment of periodontal status is mostly based on dental examination and established indices, commonly including plaque index, gingival index, bleeding on probing (BOP), periodontal probing depth (PPD), and clinical attachment loss (CAL). Some investigations also incorporate radiographic evaluations of alveolar bone loss, whereas others rely on simpler proxies such as tooth count, reported tooth loss, or self-reported periodontal status [54]. Plaque index measures the accumulation of bacterial plaque at the gingival margin, and the gingival index evaluates the color, contour, and bleeding response of the gingiva. BOP is a sensitive indicator of gingival inflammation, detected by gently probing the gingivodental sulcus and recording bleeding. More advanced periodontal destruction is captured by PPD, which quantifies the depth from the gingival margin to the base of the periodontal pocket, and by CAL, which reflects the cumulative loss of connective tissue support measured from the cementoenamel junction to the pocket base [54].

Beyond these individual measures, several classification systems have been developed to categorize the severity and extent of periodontal disease. The community periodontal index employs a 0 to 4 scale based on the occurrence of gingival bleeding, presence of calculus, PPD, and CAL. The widely adopted Center for Disease Control and prevention - American Academy of Periodontology (CDC-AAP) classification defines mild, moderate, and severe periodontitis based on the number and severity of interproximal sites with CAL and PPD. The more recent American Academy of Periodontology/European Federation of Periodontology (AAP/EFP) 2017 classification introduces a multidimensional staging (I-IV) and grading (A-C) system that accounts for disease severity, complexity, and progression risk [55].

##### Measurement approaches used in literature

In studies examining associations with carbohydrate quality or cognitive health, periodontal status can be assessed using clinical measures, including the number of teeth with PPD ≥4mm [45], the number of teeth with BOP, PPD, and/or CAL ≥4 mm, or ≥5 mm at the same site [38,56,57], or BOP to define gingivitis [40]. Administrative records can also be used to identify periodontitis cases [58]. Disease severity can be classified using CDC-AAP criteria [47,59,60], sometimes in combination with the 2017 AAP/EFP system [42,61], or using a community periodontal index ≥3 to define periodontitis [62].

#### Cognitive decline

Cognitive decline encompasses a spectrum from mild cognitive impairment (MCI) to dementia of various etiologies, including Alzheimer’s disease (AD), vascular dementia, and mixed forms [63,64]. MCI represents an intermediate state between normal aging and dementia, with affected individuals showing measurable deficits on cognitive testing but preserved functional independence [[65], [66], [67], [68]]. Although some individuals with MCI remain stable for years, others progress to dementia depending on neuropathological burden, vascular comorbidities [69], genetic risk factors such as apolipoprotein E ε4 status [70], and gender [71]. Dementia is diagnosed when independence in daily life is impaired [64]. Recognizing this continuum has led to refined diagnostic approaches that differentiate early disease stages from both normal aging and advanced neurodegeneration.

Brief cognitive screening tools such as the mini-mental state examination [72], modified mini-mental state [73], and the Montreal Cognitive Assessment [74] are widely used for initial evaluation. More comprehensive batteries, assessing episodic memory, attention, language, visuospatial skills, and executive function, are often administered longitudinally to capture trajectories of change [75]. Biomarkers, particularly amyloid-β and tau levels in cerebrospinal fluid or via Positron Emission Tomography (PET) scans, add precision by identifying early neuropathological changes, helping differentiate between normal aging and disease progression [63]. Some studies apply formal criteria for diagnosis classification. The National Institute on Aging–Alzheimer’s Association criteria [76] provide stage-specific definitions for AD with MCI and AD dementia, integrating clinical, neuropsychological, and biomarker data. The Diagnostic and Statistical Manual of Mental Disorders, fourth edition (DSM-4) [77], and fifth edition (DSM-5) [78] offer widely adopted syndromic definitions of dementia and MCI.

##### Measurement approaches used in literature

In studies examining associations between carbohydrate quality or periodontal disease and cognition, cognitive status is typically assessed using repeated neuropsychological testing (e.g., mini-mental state examination, Addenbrooke's cognitive examination (ACE), Free and cued selective reminding test (FCSRT), trail making tests, or Isaacs set test) [44,56,61]. Dementia diagnoses and subtypes are determined through clinical and imaging-based assessments, including diagnostic adjudication based on DSM or National Institute on Aging–Alzheimer’s Association criteria, neurological examinations, and neuroimaging for vascular or mixed dementia [43,56,57,61,79]. Other studies use administrative approaches, such as health claims data or International Classification of Diseases (ICD) codes, or link electronic health records with validated endpoints for all-cause dementia or AD [58,60,80,81].

### Bidirectional pathways linking refined carbohydrates, periodontal disease, and cognitive decline

**FIGURE 1:**
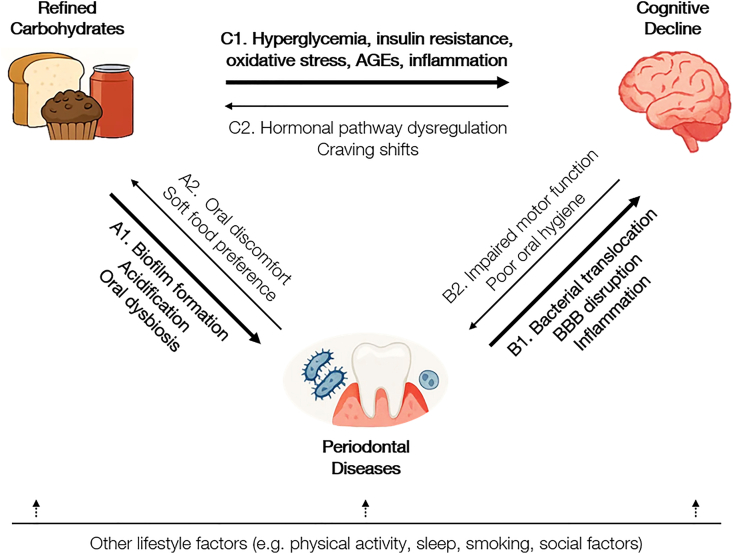
Graphical synthesis of the bidirectional interrelationships between refined carbohydrates, periodontal diseases, and cognitive decline, highlighting key underlying mechanisms. (A1) Refined carbohydrate intake promotes oral dysbiosis by enhancing dental biofilm formation, acidifying saliva, and supporting the overgrowth of pathogenic periodontal bacteria. (B1) Periodontal pathogens and the resulting systemic inflammation promote cytokine release and blood–brain barrier disruption, facilitating neuroinflammation. (C1) High intake of refined carbohydrates promotes hyperglycemia, insulin resistance, oxidative stress, and advanced glycation end-product accumulation, thereby accelerating neuroinflammation and cognitive decline. (A2) Chronic periodontal inflammation can alter taste perception, cause oral discomfort, and shift oral microbiota composition, thereby promoting a preference for sweet, soft, and easily consumed foods. (B2) Cognitive decline impairs fine motor skills and executive function, reducing oral hygiene effectiveness and facilitating the progression of periodontal disease. (C2) Cognitive impairment may dysregulate hormonal and reward pathways, intensifying cravings for sweet and refined foods.

In this section, we explore the complex, bidirectional pathways linking refined carbohydrates, periodontal diseases, and cognitive decline. Three primary pathways are most extensively supported by current literature: (Figure 1 A1) refined carbohydrate intake inducing oral dysbiosis, then periodontal diseases [[82], [83], [84]]; (Figure 1 B1) periodontitis leading to bacterial translocation, then contributing to neuroinflammation and subsequent cognitive dysfunction[[18], [19], [20]]; and (Figure 1 C1) refined carbohydrate consumption accelerating cognitive decline via metabolic dysregulations [41,46,85]. These primary axes of interaction are supported by substantial scientific literature [[18], [19], [20],^41^,^46^,[82], [83], [84], [85]].

In contrast, fewer studies have explored reverse or feedback mechanisms. These include: (Figure 1 A2) how periodontal disease can alter taste perception and cause oral discomfort leading to dietary shifts toward softer, easier to consume foods [[86], [87], [88]]; (Figure 1 B2) cognitive impairment, leading to motor function decline and thus limiting oral hygiene behaviors, contributing to the progression of periodontal disease [[89], [90], [91]]; and (Figure 1 C2) the role of neurohormonal dysregulation in cognitive decline, leading to craving shifts and eventually diet modification [[92], [93], [94]]. Collectively, these bidirectional interactions create a complex network of biological and behavioral feedback loops (Figure 1), highlighting the interdependence of dietary quality, oral health, and cognitive health.

Other lifestyle factors, such as physical activity, sleep quality, smoking, and social determinants of health, might further impact refined carbohydrate consumption and oral and cognitive health.

Studies used to discuss each bidirectional relationship were identified through structured searches of PubMed and Web of Science using combinations of keywords related to carbohydrate quality, periodontal disease, and cognitive decline. Mainly epidemiological studies, meta-analyses, and reviews published over the past 10 y in adult human populations were selected, with emphasis on recent high-quality longitudinal cohort studies. Studies focusing on specific populations, including pregnant women, athletes, individuals with obesity, or those with diagnosed metabolic or chronic diseases, were avoided to focus on the general adult population. Studies relying exclusively on animal models or in vitro data were not included as primary evidence but are occasionally referenced to illustrate potential biological mechanisms. When interpreting findings, particular attention was given to study design, exposure and outcome assessment methods, and adjustment for major confounding factors.

#### Refined carbohydrates and periodontal diseases: a bidirectional relationship

Recent reviews have highlighted consistent associations between high intakes of sucrose, fructose, or free sugars and increased risk of periodontal disease, partially explained by systemic low-grade inflammation and disruption of the oral microbiota [[82], [83], [84]]. More specifically, frequent consumption of added sugars or SSBs has been linked to a higher risk of periodontitis across age groups, including adults [42], teenagers [38], and children [40]. In contrast, higher intakes of whole grains have been associated with better periodontal outcomes [45], and a lower carbohydrate-to-fiber ratio (indicating greater fiber intake) has been significantly associated with reduced odds of moderate-to-severe periodontitis [47].

Mechanistically, diets high in refined carbohydrates promote rapid postprandial hyperglycemia, which lowers salivary pH, fosters cariogenic and periodontopathogenic biofilm formation [95], and directly fuels the growth of bacteria such as *P. gingivalis* and *Streptococcus mutans* [82,96,97]. In parallel, repeated glycemic fluctuations trigger systemic insulin resistance and low-grade inflammation, both of which impair periodontal tissue resilience through increased cytokine release (e.g., IL-6, TNF-α) and impaired vascularization [11,98]. Hyperglycemia also enhances the formation of advanced glycation end-products (AGEs), which accumulate in gingival tissues, interact with receptors of AGEs, and amplify oxidative stress and inflammatory signaling cascades [99].

On the contrary, periodontal inflammation and oral dysbiosis may also influence dietary behavior. Inflammation and microbial imbalance can alter taste perception and enhance preference for sweet or refined foods, potentially reinforcing a cycle of dysbiosis and disease. Emerging research suggests that changes in oral microbiota can modify taste sensitivity and thus influence food choices [88], with preliminary evidence linking microbial composition to altered taste thresholds driving dietary shifts [87]. Additionally, periodontitis has been shown to impair masticatory performance [100], leading individuals to avoid hard-to-chew foods such as raw vegetables, fruits, or nuts. Tooth loss, in particular, has been associated with lower intake of fiber-rich foods and vegetables [86,101]. These changes can foster a preference for soft, easy-to-chew foods, such as juices, mashed potatoes, and cakes, which are frequently refined and less nutrient-dense [101].

Periodontal disease and type 2 diabetes, which is characterized by insulin resistance and often aggravated by refined carbohydrate intake, share a bidirectional relationship that amplifies systemic inflammation and glycemic dysregulation, processes that may ultimately impair cognitive outcomes [102].

Taken together, literature points to a bidirectional relationship. Diets high in refined carbohydrates increase susceptibility to periodontal disease through microbial and metabolic mechanisms, while periodontal inflammation and impaired oral function may promote shifts toward softer, more refined foods, creating a vicious cycle.

#### Periodontal diseases and cognitive decline: a bidirectional relationship

A growing number of reviews suggest a link between periodontal disease and cognitive disorders, mediated primarily by systemic inflammation and bacterial endotoxins that can cross the blood–brain barrier (BBB) [[18], [19], [20]]. Longitudinal studies further support this link, with periodontitis associated with a higher risk of cognitive decline and dementia in diverse populations, including over 6 y in Turkey [57, 8[57], 8 y in Taiwan [58], and across a 15-y period in the United States [61]. Meanwhile, improved oral health and chewing abilities seem to be preventive for dementia [103].

Mechanistically, periodontal disease is characterized by a persistent inflammatory response, often driven by pathogens such as *P. gingivalis* [82]. This bacterium produces gingipains and lipopolysaccharides, which can translocate into systemic circulation, compromise BBB integrity, and accumulate in the brain [18,[104], [105], [106], [107]]. Postmortem studies have confirmed the presence of gingipains in individuals with dementia [[104], [105], [106]]. Once in the brain, these bacterial products initiate microglial activation, oxidative stress, and the aggregation of amyloid-β and tau proteins, thereby accelerating neurodegeneration [18,107]. Systemic inflammatory mediators (e.g., IL-1β, IL-6, and TNF-α) triggered by the periodontal pathogens further contribute by amplifying both peripheral tissue destruction and central neuroinflammatory processes [82,107]. Sustained inflammation of periodontal origin has been directly associated with a greater risk of AD and other dementias, in part through mechanisms involving BBB disruption, microglial overactivation, and oxidative injury [17]. Recent findings suggest additional molecular and vascular pathways linking periodontal and neuroinflammation [108]. Periodontal microRNAs detectable in gingival crevicular fluid may modulate inflammatory responses [22] and contribute to AD by influencing mitochondrial function, amyloid-β accumulation, tau phosphorylation, and neuroinflammation [23]. In parallel, periodontal treatment has been shown to improve markers of vascular endothelial function [24] and reduce cerebrovascular risk [109], supporting a vascular-mediated pathway from periodontal inflammation to cognitive decline [25]. Moreover, chronic periodontal inflammation can promote metabolic dysfunction. Elevated inflammatory and tissue-degrading mediators contribute to insulin resistance [49,110], disrupt mitochondrial function, and enhance oxidative stress, providing an indirect metabolic pathway linking periodontitis to cognitive decline [49,98]. More specifically, oxidative stress contributes directly to periodontal tissue destruction, as chronic periodontal inflammation promotes the accumulation of reactive oxygen species that damage lipids, proteins, and DNA [111]. This oxidative imbalance is reflected systemically, with studies reporting reduced total antioxidant capacity and elevated oxidative stress markers in individuals with both periodontitis and AD, supporting a mechanistic link between periodontal pathology and cognitive decline via oxidative stress pathways [112].

A bidirectional relationship between cognitive decline and periodontitis is also suggested in the literature, where cognitive decline may worsen periodontal health, creating a feedback loop [19]. As dementia progresses, cognitive impairments, such as declines in executive function, memory, and motor skills, make it increasingly difficult for individuals to maintain oral hygiene. Reviews report significantly poorer oral health in dementia patients compared with cognitively healthy individuals [90,91]. Common oral issues in dementia include elevated plaque levels, gingival bleeding, periodontal pockets, stomatitis, mucosal lesions, and reduced salivary flow [91]. This deterioration is further exacerbated by a decreased use of dental services in dementia patients, particularly as the disease advances [89].

Altogether, current research suggests that periodontal disease may contribute to neurodegeneration through inflammatory and bacterial pathways, as well as insulin resistance, while cognitive decline can further impair oral health through diminished oral hygiene and reduced dental service access.

#### Refined carbohydrates and cognitive decline: a bidirectional relationship

Recent studies show growing interest in the role of glycemic fluctuations and sugar intake in cognitive aging. A meta-analysis of longitudinal data reported a trend linking greater glycemic variability to increased AD risk, though results were not consistently statistically significant [85]. More consistently, associations have been found between SSB consumption and cognitive disorders in a recent review [41], as well as between high intake of total sugars, particularly fructose and sucrose, and accelerated cognitive decline and dementia risk over 5 y [39]. Similarly, high-sugar diets and high absolute or relative sugar intake were significantly associated with increased risk of all-cause dementia and AD over an 11-y follow-up [80]. Specific dietary patterns may also matter: high-glycemic load from afternoon snacks was associated with poorer visual, episodic, and global cognition, and elevated dementia risk in carriers [43,44]. The type of sugar also appears important: intake of free sugars, but not intrinsic free sugars (e.g., whole fruits), was linked to dementia risk [37], and although sugar-sweetened and artificially sweetened beverages were associated with increased AD risk, moderate consumption of pure fruit or vegetable juices showed potential protective effects [81]. Conversely, nonstarch polysaccharides (e.g., fibers) appear to offer neuroprotective effects by reducing neuroinflammation, supporting synaptic integrity, and modulating gut microbiota [46].

Mechanistically, high intake of refined carbohydrates and resultant glycemic variability initiate a cascade of metabolic stressors that contribute to neurodegeneration. Chronic hyperglycemia impairs cerebral glucose metabolism, promotes insulin resistance, and generates AGEs, which interact with neuronal Receptor for Advanced Glycation Endproducts (RAGEs) to amplify oxidative stress and neuroinflammation [11,85,99]. Poor vascularization of the brain, driven by hyperglycemia-induced capillary damage, further compromises nutrient delivery and accelerates neurodegeneration [113]. Fructose, metabolized independently of insulin, further drives hepatic lipogenesis, hepatic insulin resistance, and oxidative stress [114]. In parallel, high-glycemic diets elevate circulating free fatty acids and proinflammatory cytokines, reflected in increased C-reactive protein (CRP), sustaining systemic and central inflammation [99,[115], [116], [117], [118], [119]]. Additionally, cerebral insulin resistance disrupts neuronal insulin signaling, impairs mitochondrial energy metabolism, and compromises synaptic function, rendering neurons more vulnerable to oxidative damage and excitotoxicity [120]. Emerging evidence suggests that AD and type 2 diabetes share multiple pathophysiological features, including insulin resistance, oxidative stress, neuroinflammation, mitochondrial dysfunction, and AGEs, supporting the characterization of AD as a form of “type 3 diabetes” [121]. Together, these interconnected pathways compromise synaptic function and accelerate cognitive decline.

Cognitive impairment may also influence dietary behavior, further reinforcing a feedback loop. Neurodegeneration disrupts appetite regulation and reward processing via the leptin–ghrelin axis and dopaminergic pathways, often leading to heightened preference for sweet, energy-dense foods. Altered eating behaviors in dementia include changes in meal frequency and quantity, persistent ingestion of nonnutritive substances (pica), and, most commonly, increased preference for sweet foods [92,94,122]. This symptom is especially frequent in frontotemporal dementia, where sweet cravings are reported in the vast majority of patients, and to a lesser extent in AD [93].

Once again, a bidirectional relationship is suggested in the literature: diets rich in refined carbohydrates are associated with increased risk of cognitive decline, whereas fiber-rich and unrefined foods appear protective. In turn, cognitive impairment (though to a lesser extent) may contribute to dietary shifts toward sweeter and more refined foods, through disruptions in appetite regulation and hormonal pathways.

### Integrative mechanisms

The interrelationships between refined carbohydrate intake, periodontal diseases, and cognitive decline are mediated by a network of overlapping biological and behavioral mechanisms. Among these, insulin resistance, chronic inflammation, oxidative stress, and microbiota dysregulation serve as key integrative mechanisms.

Insulin resistance [48,49,123], chronic inflammation [21,83,107], and oxidative stress [99,124] are all central shared pathways across all 3 dyads. Rather than functioning in isolation, these mechanisms interact, creating self-reinforcing cycles that could accelerate both periodontal pathology and neurodegenerative processes. Dietary patterns rich in antioxidants, in turn, can attenuate this oxidative burden, and anti-inflammatory diets contribute to the reduction of periodontal and neuro-inflammation, which may explain the protective associations observed between diets emphasizing fruits, vegetables, and whole grains, and a lower risk of both periodontitis and cognitive decline [125,126].

Refined carbohydrates and periodontitis synergistically disrupt oral and gut microbiota, leading to increased gut permeability, BBB disruption, and translocation of bacterial metabolites into the circulation and eventually to the brain, where they can trigger or amplify neuroinflammatory responses [127,128]. Conversely, diets rich in fiber, whole grains, fruits, vegetables, and healthy fats support microbial diversity, preserve gut barrier integrity, lower inflammation, and are linked to better oral and cognitive outcomes [47,125,[129], [130], [131]]. A key mechanism involves the fermentation of nondigestible carbohydrates such as fibers into short-chain fatty acids, which improve glycemic control and sustain microbiota balance [132,133].

Gut microbiota influence insulin resistance through microbial metabolism of carbohydrates and modulation of the gut-brain-metabolic axis, which affects both metabolic regulation and cognitive function [134]. Gut and oral microbiota dysbiosis in AD contributes to neuroinflammation by promoting endotoxin translocation, activating microglia through Toll-like receptor 4 (TLR4) pathways, and altering metabolite signaling, while certain microbial products, such as butyrate, may exert protective anti-inflammatory effects [130]. Integrating these mechanisms underscores the importance of addressing diet, oral health, and cognition simultaneously.

### Perspectives and public health implications

The discussed findings highlight 3 consistent bidirectional patterns linking diet, oral health, and cognition. First, highly refined carbohydrate intake was associated with oral dysbiosis and periodontitis, whereas periodontal disease itself appeared to influence food choices toward softer, more carbohydrate-dense options. Second, periodontitis was linked to neuroinflammation and subsequent cognitive decline, whereas cognitive impairment contributed to poorer oral hygiene and progression of periodontal disease. Third, frequent consumption of refined carbohydrates was related to metabolic disturbances and accelerated cognitive decline, whereas cognitive deterioration was associated with neurohormonal changes that may further shift dietary behaviors toward sweeter and more refined dietary patterns.

#### Knowledge gaps and research priorities

Despite growing evidence linking refined carbohydrates, periodontal health, and cognitive outcomes dyadically, important gaps remain. No study has yet comprehensively examined this tripartite relationship, limiting our understanding of shared mechanisms and potential mediating pathways. The bidirectionality of these associations also remains poorly understood, raising critical questions about feedback loops. Undisputed terminology and standardization of measurement methods for all 3 factors are essential for scientifically rigorous advances in this complex field.

A life-course perspective is equally important. Few studies have investigated how early dietary exposures shape both oral and cognitive trajectories. However, studies suggest that taste preferences established in utero or during breastfeeding may predispose individuals to long-term consumption of energy-dense, nutrient-poor foods [135], and that consistent exposure to refined carbohydrates may affect neurodevelopment, potentially influencing metabolic health, and cognitive reserve later in life, accentuating vulnerability to neurodegeneration [136]. Future research should therefore move beyond dyadic associations to explore biological pathways, including glycemic variability, oxidative stress, systemic inflammation, insulin resistance, and oral and gut microbiota dysbiosis. Longitudinal and mechanistic studies that integrate dietary, oral, and cognitive assessments are urgently needed. Such efforts will require interdisciplinary collaboration between nutritionists, dentists, neurologists, epidemiologists, and policymakers.

#### Social and lifestyle determinants

Scientific progress must also be matched with recognition of the social realities that shape health across the lifespan. Socioeconomic status strongly influences dietary quality, oral care access, and cognitive reserve, reinforcing health inequities. Low socioeconomic status is associated with poorer diet quality, as shown in a large United States cohort study [137]. Food insecurity has been associated with accelerated cognitive decline, particularly affecting executive function [138], whereas structural barriers to dental care contribute to poorer oral health outcomes [139]. Gender and social inequalities also impact cognitive reserve, with women in low- and middle-income countries being particularly vulnerable due to compounded socioeconomic disadvantages [140]. Early-life socioeconomic adversity also predicts later cognitive impairment, underscoring the long-term impact of structural determinants [141].

These findings demonstrate that oral and cognitive health cannot be understood in isolation from their social context, and highlight why prevention strategies must move beyond single interventions. Interdisciplinary approaches that combine dietary improvements and oral hygiene education, but also consider other lifestyle factors such as physical activity [142], sleep quality [143], smoking behavior [144], cognitive stimulation [145], and social engagement [146], are to be considered for synergic benefits in reducing dementia risk. Multidomain lifestyle interventions have emerged as promising strategies to prevent or delay cognitive decline [[147], [148], [149], [150]]. Together, these results make clear that addressing dementia requires attention not only to biological mechanisms but also to the environments and resources that shape health behaviors throughout life.

#### Public health implications

Given the rising global burden of dementia and the largely symptomatic nature of available treatments, prevention must become a central public health priority [64]. Evidence on the oral–gut–brain axis indicates that oral microbiome dysbiosis, driven by consistent refined carbohydrate intake, contributes to systemic inflammation and neuroinflammation, reinforcing the interconnected role of diet and oral health in brain aging [127]. Carbohydrate quality, therefore, represents a critical policy lever. Substituting refined grains with whole grains and increasing dietary fiber intake are effective strategies to improve both oral and cognitive outcomes [8,[151], [152], [153]]. More broadly, policies should aim to limit access to refined foods while enhancing access to whole, nutrient-dense foods such as fruits, vegetables, legumes, and nuts to promote healthy aging [154,155]. Fiscal and regulatory measures such as sugar taxes, subsidies for healthy foods[156], front-of-package warnings [157], and restrictions on marketing unhealthy products to children [158] yield substantial health benefits by shaping healthier dietary habits [159] and should therefore be expanded.

Oral health requires equally urgent policy attention. Despite being largely preventable, oral diseases remain widespread among older adults due to inadequate access to routine care, reflecting persistent gaps in policy frameworks [160]. Expanding access to preventive services, integrating oral health into primary care, and providing caregiver education are cost-effective measures that could meaningfully reduce dementia risk [[161], [162], [163]]. Along these lines, public health policies should address antimicrobial resistance and oral microbiota disruption caused by antibiotic overuse [164,165], by promoting, for example, combined probiotic-antibiotic approaches [166,167]. Given their shared mechanisms, aligning dietary and oral health policies offers an efficient and synergistic approach to dementia prevention.

Finally, effective prevention must be framed within a multidomain and equity-driven agenda. Beyond diet and oral health, policies should foster environments that promote, for instance, physical activity [168], sleep quality [169], lifelong learning [170], and social inclusion [146], while reducing the structural barriers that perpetuate inequalities [171], to provide enduring resilience against dementia. Population-wide strategies that integrate biological, behavioral, and social dimensions are likely to achieve the greatest impact and ensure more equitable dementia prevention at scale.

In conclusion, the interrelationships between refined carbohydrate intake, periodontal disease, and cognitive decline are mediated by overlapping behavioral and biological mechanisms, including insulin resistance, chronic inflammation, oxidative stress, and microbiota dysregulation. These pathways interact within self-reinforcing cycles rather than acting independently. High intake of refined carbohydrates promotes oral dysbiosis and metabolic disturbances that increase susceptibility to periodontal disease and neurodegenerative processes, whereas periodontal inflammation may further amplify these effects through bacterial translocation and blood–brain barrier disruption. Reverse mechanisms may also contribute, as oral discomfort and neurohormonal changes drive preferences for sweeter, more processed foods, while cognitive impairment can compromise motor function and thus oral hygiene.

Future longitudinal and mechanistic studies examining these 3 factors simultaneously are needed to clarify shared pathways and mediating mechanisms and to identify strategies to reduce the growing burden of refined carbohydrate consumption, oral disease, and cognitive decline. These relationships unfold within broader social and behavioral contexts, as socioeconomic conditions, access to oral health care, and lifestyle factors shape diet quality, oral health, and cognitive reserve across the life course. Integrating nutritional policies, oral health promotion, and multidomain lifestyle interventions may therefore offer a promising approach to reduce dementia risk and support healthier aging at the population level.

## Author contributions

The authors’ responsibilities were as follows – SA, CB, MR: designed research; DR: wrote the first draft; SA, CB, MR: reviewed and edited the final manuscript. DR, SA, CB, MR: had primary responsibility for final content; and all authors: read and approved the final manuscript.

## Data availability

For the purpose of open access, a Creative Commons Attribution (CC-BY) public copyright license has been applied by the authors to the present document and will be applied to all subsequent versions up to the author-accepted manuscript arising from this submission.

## Funding

This work was supported by the French National Research Agency, ANR DentalCog, ANR-22-CE36-0003-01. The funding source had no role in study design, data collection, analysis, interpretation, manuscript writing, or the decision to submit for publication.

## Declaration of generative AI and AI-assisted techologies in the writing process

The authors declare that no generative AI or AI-assisted technologies were used in the writing of this manuscript.

## Conflict of interest

The authors report no conflicts of interest.
